# The Modulation of Immune Responses in *Tilapinevirus tilapiae*-Infected Fish Cells through MAPK/ERK Signalling

**DOI:** 10.3390/v15040900

**Published:** 2023-03-31

**Authors:** Tuchakorn Lertwanakarn, Matepiya Khemthong, Puntanut Tattiyapong, Win Surachetpong

**Affiliations:** 1 Department of Physiology, Faculty of Veterinary Medicine, Kasetsart University, Bangkok 10900, Thailand; fvettol@ku.ac.th; 2 Department of Veterinary Microbiology and Immunology, Faculty of Veterinary Medicine, Kasetsart University, Bangkok 10900, Thailand

**Keywords:** tilapia lake virus, mitogen-activated protein kinase/extracellular signal-regulated kinase, viral replication, fish cells

## Abstract

Tilapia lake virus (TiLV) is a novel RNA virus that has been causing substantial economic losses across the global tilapia industry. Despite extensive research on potential vaccines and disease control methods, the understanding of this viral infection and the associated host cell responses remains incomplete. In this study, the involvement of the mitogen-activated protein kinase/extracellular signal-regulated kinase (MAPK/ERK) pathway in the early stages of TiLV infection was investigated. The results showed a distinct pattern of ERK phosphorylation (p-ERK) upon TiLV infection in two fish cell lines, E-11 and TiB. Specifically, the p-ERK levels in the TiB cells decreased substantially, while the p-ERK levels in the E-11 cells remained constant. Interestingly, a large number of cytopathic effects were observed in the infected E-11 cells but none in the infected TiB cells. Furthermore, when p-ERK was suppressed using the inhibitor PD0325901, a significant reduction in the TiLV load and decrease in the *mx* and *rsad2* gene expression levels were observed in the TiB cells in days 1–7 following infection. These findings highlight the role of the MAPK/ERK signalling pathway and provide new insights into the cellular mechanisms during TiLV infection that could be useful in developing new strategies to control this virus.

## 1. Introduction

The outbreaks of tilapia lake virus (TiLV), or *Tilapinevirus tilapiae*, have caused significant morbidity and mortality among the tilapia population in many countries, leading to economic and social impacts on farmers who rely on tilapia aquaculture [1,2,3]. TiLV is a negative-sense single-stranded RNA virus that is currently classified under the family *Amnoonviridae* [4]. Infection with TiLV is associated with a range of clinical signs in fish, including abnormal swimming, exophthalmos, a swollen abdomen, severe anaemia and multiple skin hemorrhages [5,6,7]. The virus also causes macroscopic alterations in the internal organs of infected fish, such as a pale and enlarged liver, a contracted spleen, anterior kidney and intestinal edema [5,7,8]. The microscopic changes in the internal organs of TiLV-infected tilapia include lymphocytic infiltration and multiple areas of necrosis in the liver, spleen, anterior kidney and brain [5,6,7,8,9]. In addition, experimental studies have shown that TiLV induces cell death in various piscine cell lines [5,10,11,12,13]. The mechanisms of cytotoxicity are therefore thought to be associated with the disruption of mitochondrial function [6]. Despite many strategies aimed at controlling this viral infection, including good farming practices [2], the application of probiotics [14,15], antiviral therapy [16] and vaccine production [17,18,19], the pathogenesis of the host–viral interaction is still poorly understood, and the detailed mechanism of intracellular signalling underlying TiLV infection remains unclear.

The mitogen-activated protein kinase (MAPK) cascade is a fundamental signalling pathway in animals which regulates various cellular processes, including differentiation, proliferation and survival [20]. This cascade is initiated by extracellular stimuli such as growth factors, hormones, signals and pathogens, and further activates downstream MAPK proteins such as MAPK kinase kinase (MAPKKK), MAPK kinase (MAPKK) and MAPKs [21]. In fish, there are three branches of the MAPK cascade: extracellular signal-regulated kinase (ERK), c-Jun terminus N-terminus kinase (JNK) and p38 pathways, which play distinct roles in regulating cellular responses. In tilapia, heat stress [22], high salinity [23] and low oxygen [24] can promote MAPK signaling as its adaptation process in response to these extreme environments. Additionally, infection by *Streptococcus agalactiae* in tilapia could also upregulate raf/MEK/ERK and p38 MAPK to stimulate the host immune response to fight the bacteria [25,26].

Generally, the ERK pathway, or raf/MEK/ERK signalling, is a crucial cascade in the cellular response to viral infections. Previous studies have demonstrated that many viruses, such as coronavirus, coxsackievirus, human immunodeficiency virus, the influenza virus and the hepatitis C virus, utilise this ERK pathway in viral entry mechanisms, the replication and regulation of host gene expression and the production of antiviral and proinflammatory cytokines [27,28,29,30,31,32,33]. However, studies of these mechanisms and the involvement of MAPK/ERK signalling in the pathogenesis and replication of piscine viruses are still limited, especially with respect to TiLV infection. To address this knowledge gap, we investigated the role of MAPK/ERK signalling in TiLV-infected fish cell lines. Findings revealed the downregulation of MAPK/ERK during early TiLV infection and thus highlight the importance of this pathway in the regulation of viral RNA copies and the expression of immune-related genes.

## 2. Materials and Methods

### 2.1. Cell Culture and Virus Infection

To investigate the role of MAPK/ERK signalling, we used two fish cell lines: the E-11 cell line, which originates from snakehead fry, and tilapia brain cells (TiB). The E-11 cell line was obtained from the European Collection Authenticated Cell Cultures (ECACC), and the TiB was isolated from the brains of healthy red hybrid tilapia (*Oreochromis* spp.) following a previously described protocol [34]. Both cell lines (n = 3 per group) were cultured in Leibovitz’s L-15 media supplemented with 2 mM glutamine and 10% fetal bovine serum (FBS) at 25 °C without CO_2_. The virus was inoculated into the cells at a multiplicity of infection of 0.1 for 1 h, after which the media were removed. The morphological changes in the inoculated cells were observed at 3, 6, 24, 48 and 72 h post-inoculation (hpi). The cells were then harvested via scraping in line with an established protocol [16] and stored at −80 °C until analysis.

### 2.2. Inhibition of MAPK-ERK Signalling

A vial containing 5 mg of PD0325901 (Cell Signaling Technology, Danvers, MA, USA) was dissolved in 1.03 mL dimethyl sulfoxide (DMSO, Sigma-Aldrich, St Louis, MO, USA) to produce a 10 mM stock. The stock solution was serially diluted to create final concentrations of 30, 10, 3, 1 and 0.3 µM using Leibovitz’s L-15 media supplemented with 5% FBS. To inhibit the ERK pathway, the cells were pretreated with various PD0325901 concentrations for 1 h prior to TiLV inoculation.

### 2.3. Western Blot Analysis

For the protein and phosphoprotein analysis, the cells were collected in a lysis buffer (50 mM potassium phosphate buffer, 400 mM NaCl, 100 mM KCl, 10 mM imidazole, 10% glycerol and 0.5% Triton-X100; pH 7.8). After thawing, the lysates were manually homogenised, and the supernatant was collected to determine the protein concentration using a BCA assay kit (Pierce^TM^, Thermo Fisher Scientific, Rockford, IL, USA). Samples were then loaded onto 10% SDS-PAGE and electrophoresed at 200 V for 45 min. The gels were transferred onto 0.45 µm PVDF membranes (Immuno-Blot^®^, BioRad, Hercules, CA, USA), and non-specific proteins were blocked with 3% bovine serum albumin (Merck, Darmstadt, Germany) in a phosphate-buffered saline-Tween solution (136 mM NaCl, 10.14 mM Na_2_HPO_4_, 2.6 mM KCl, 1.76 mM KH_2_PO_4_, 0.1% [*v*/*v*] Tween-20; pH 7.5). The alteration in MAPK/ERK phosphorylation was studied by incubating the membranes overnight with primary antibodies for p-p44/42 (Cell Signaling Technology, Danvers, MA, USA), total p44/42 (Cell Signaling Technology, Danvers, MA, USA) and β-actin (Clone 4, Darmstadt, Germany). Secondary antibodies (goat anti-rabbit; Cell Signaling Technology, Danvers, MA, USA) were added and incubated for 1 h. The blots were developed using enhanced chemiluminescence (Lumiflash™, Visual Protein, Taiwan) and imaged using a ChemiDoc™ MP imager (BioRad, USA). The band densities were analysed using ImageLab™ 6.1 software (BioRad, USA). All the phosphorylated bands were normalised to the total protein and β-actin.

### 2.4. RNA Extraction, cDNA Construction and qPCR

The RNA extraction procedure was performed as previously described [16]. Briefly, cell lysates were combined with a Trizol reagent (GENEzol^TM^, Geneaid, Taiwan) and chloroform (Sigma-Aldrich, USA) and incubated for 3 min. The mixture was then centrifuged at 12,000× *g*, 4 °C for 15 min. The supernatant was collected and mixed with 1 µL of DNase I (Thermo Fisher Scientific, Carlsbad, CA USA) and incubated at 37 °C for 30 min, after which 2-propanol (Merck, Darmstadt, Germany) was added. The mixture was then stored at −20 °C for 2 h. After thawing, the lysates were centrifuged at 12,000× *g*, 4 °C for 15 min, and the supernatant was discarded. The pellet was washed with 1 mL of 75% ethanol twice and air-dried. The RNA pellet was resuspended in 20 µL of diethylpyrocarbonate (DEPC)-treated water. The amount of RNA was measured using a NanoDrop spectrophotometer (NanoDrop2000, Thermo Fisher Scientific, Wilmington, DE, USA). The RNA pellet was converted to cDNA using ReverTraAce™ qPCR RT MasterMix (Toyobo, Osaka, Japan) in accordance with the manufacturer’s instructions. Then, an SYBR green-based RT-qPCR assay was performed to determine the TiLV viral copy [35]. The cycling conditions were initially set at 98 °C for 3 min, followed by 40 cycles at 95 °C for 10 s, 60 °C for 30 s and finally, heating to 95 °C. The melting temperature was calculated using the software. The log copy number of the TiLV virus was extrapolated from the standard curve, and the expressions of the *mx*, *rsad2*, *il-8* and *il-1β* genes were calculated using 2^−ΔΔCt^ and normalised to the housekeeping β-actin gene. The forward and reverse primers of the studied genes are provided in the supplementary files (Table S1).

### 2.5. Statistical Analysis

We conducted the statistical analyses for this study using Prism™ software (GraphPad Software, San Diego, CA, USA). The normal distribution of data was assessed using the Shapiro–Wilk test, and all the data was found to be normally distributed. A two-way ANOVA followed by Fisher’s exact test were used to compare the amount of viral RNA, the normalised band intensity obtained from the Western blot study and the normalised gene expression from the qRT-PCR between the groups. The data are represented as means ± SEMs, and statistical significance was established as *p* < 0.05.

## 3. Results

### 3.1. Different Cell Responses to TiLV Infection in E-11 and TiB Cells

**Figure 1:**
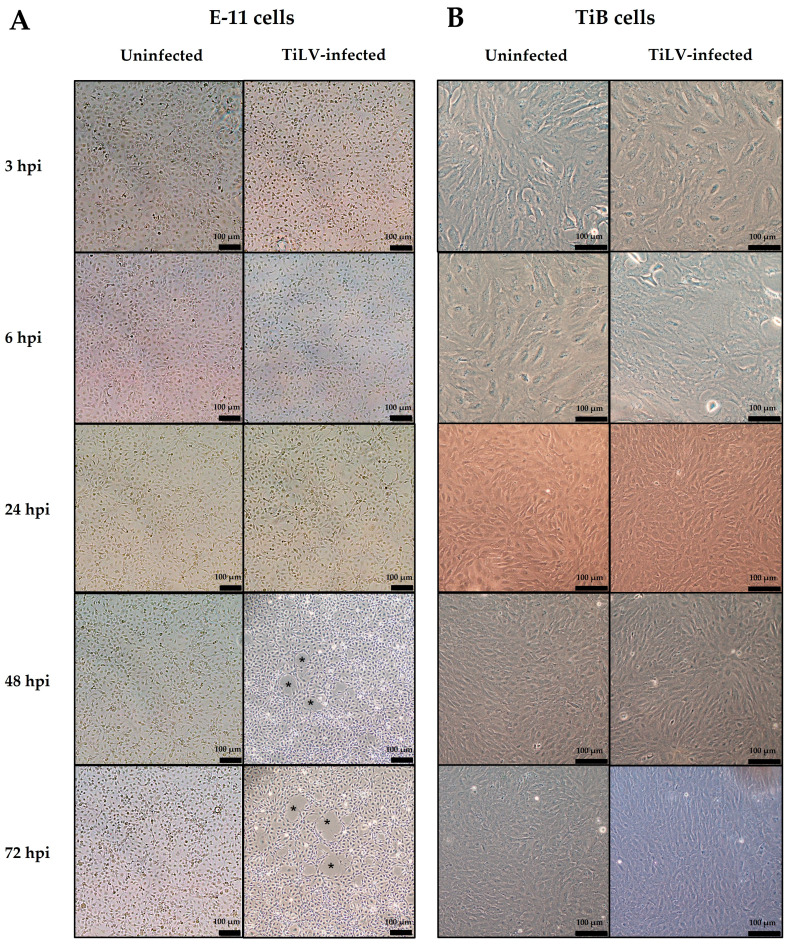
Representative figures of uninfected and tilapia lake virus (TiLV)-infected cells at different time points post-infection are presented in (**A**) the E-11 cells and (**B**) TiB cells. TiLV induced CPE formation (asterisks) in the E-11 cells at 48 and 72 hpi, while no morphological changes were observed in the TiB cells at any of the examined time points.

**Figure 2:**
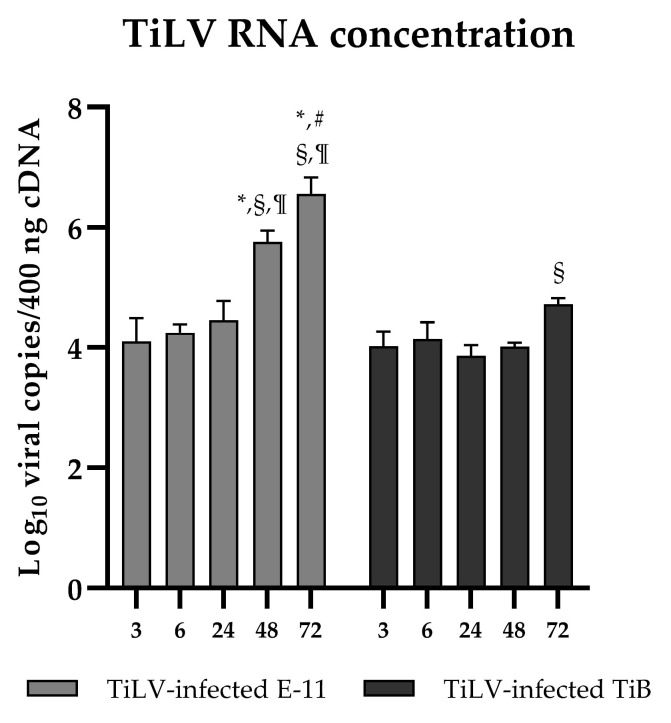
The number of TiLV RNA copies in the E-11 and TiB cells was determined following TiLV infection (n = 3 each) and is presented as the mean ± SEM. The statistical analysis revealed significant differences at various time points, as indicated by the following: * *p* < 0.05 compared to 3 hpi, # *p* < 0.05 compared to 6 hpi, § *p* < 0.05 compared to 24 hpi and ¶ *p* < 0.05 compared to 48 hpi.

The morphology of the E-11 and TiB cells was evaluated at different time points following TiLV inoculation (Figure 1). Cytopathic effects (CPEs) were observed in the infected E-11 cells at 48 hpi and 72 hpi (Figure 1A), while no morphological changes were found in the TiLV-infected TiB cells until 72 hpi (Figure 1B). The viral RNA concentration was measured in both the infected E-11 and TiB cells simultaneously (Figure 2). The viral RNA concentration in the infected E-11 cells increased significantly from 3 to 72 hpi, with a range from 4.10 ± 0.39 to 6.56 ± 0.27 copies, while the viral RNA concentration in the infected TiB cells gradually increased from 4.03 ± 0.24 at 3 hpi to 4.73 ± 0.09 copies at 72 hpi (Figure 2). Notably, a significant increase in viral copies was observed at 72 hpi compared to 24 hpi (3.87 ± 0.18 copies) (Figure 2). These findings indicated TiLV-induced cytotoxicity in the E-11 cells but no CPEs in the TiB cells.

### 3.2. Inihibition of ERK Phosphorylation in TiB Cells following TiLV Infection

**Figure 3:**
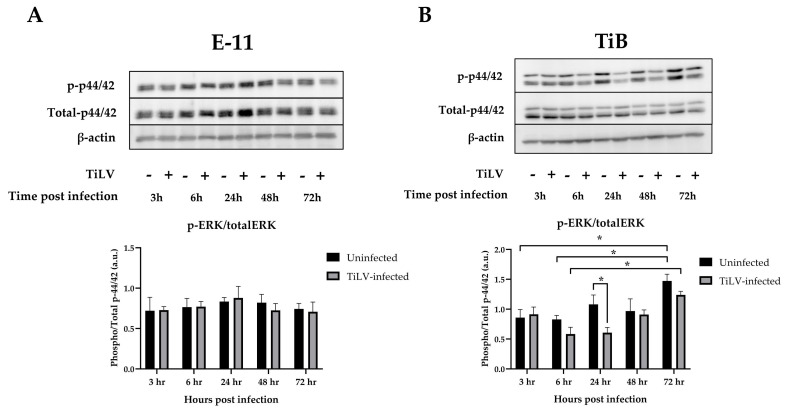
The determination of ERK phosphorylation (p-ERK) in (**A**) the E−11 cells and (**B**) TiB cells following TiLV infection (n = 3 each). The results demonstrate a significant suppression of p-ERK in the TiB cells at 24 hpi (**B**). The data are presented as means ± SEMs with * *p* < 0.05.

To investigate the involvement of the MAPK/ERK signalling pathway in TiLV infection in the E-11 and TiB cells, the phosphorylation of ERK1/2 (p-ERK) was evaluated and is presented in Figure 3. The results demonstrated no change in the p-ERK levels in the E-11 cells regardless of TiLV infection (Figure 3A). In contrast, the TiB cells exhibited a progressive increase in p-ERK levels, with a significant increase observed at 72 hpi (1.47 ± 0.12) compared to 3 hpi (0.86 ± 0.14) and 6 hpi (0.83 ± 0.07) (Figure 3B). Notably, p-ERK was suppressed at 24 hpi in the TiLV-infected TiB cells (0.61 ± 0.09) compared to the uninfected cells (1.08 ± 0.16) (Figure 3B), which suggests the potential involvement of the MAPK/ERK signalling pathway during the early stages of the TiLV infection process in the TiB cells. Furthermore, a progressive expression of p-ERK from 6 hpi to 72 hpi was also observed in TiLV-infected TiB cells (Figure 3B).

### 3.3. Suppression of ERK-Signalling Reduces Viral Copies and Altered Immune-Related Genes during Early TiLV Infection

**Figure 4:**
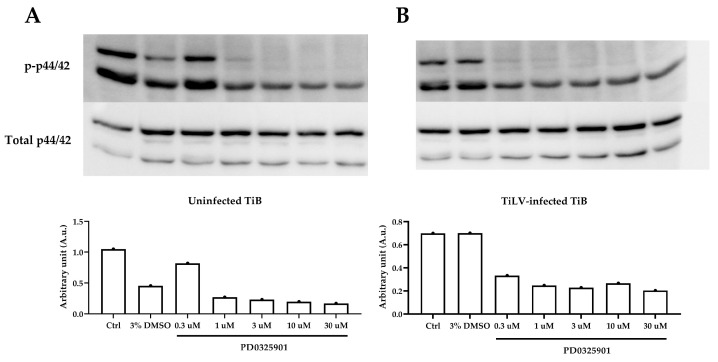
Evaluation of p-ERK (p-p44/42) in (**A**) the uninfected TiB cells and (**B**) TiLV-infected cells using PD0325901 (PD) at concentrations of between 0.3–30 μM. The band intensity of p-p44 was completely faded when the TiB cells were incubated with between 10–30 μM of PD.

To further investigate the role of the MAPK/ERK signalling pathway in TiLV infection, we examined the effects of pretreatment with PD0325901 (PD) on the p-ERK levels in the TiB cells before and after infection with TiLV (Figure 4). In the uninfected TiB cells, PD significantly suppressed p-ERK at concentrations of between 1–30 µM (Figure 4A). Similarly, in the TiLV-infected TiB cells pretreated with PD, the p-ERK levels were reduced in a dose-dependent manner at concentrations of between 0.3–30 µM (Figure 4B). Notably, complete inhibition of p-p44 was observed in the TiB cells pretreated with between 3–30 µM of PD in both conditions.

**Figure 5:**
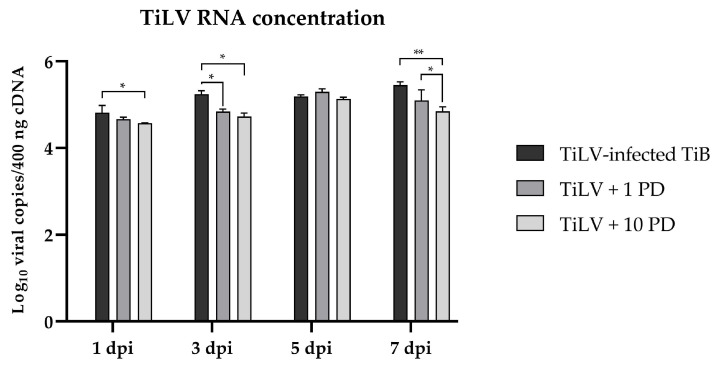
Comparison of TiLV RNA copies in TiB cells exposed to TiLV and PD, TiLV with 1 μM PD and TiLV with 10 μM PD (n = 3 each). The data are presented as means ± SEMs. * *p* < 0.05, ** *p* < 0.01.

The results of inhibiting the ERK signal using PD at low (1 µM) and high (10 µM) concentrations to determine the effects on the TiLV concentrations revealed that pretreatment with a low PD concentration significantly reduced the viral RNA copies at 3 days post-infection (dpi) (4.84 ± 0.05) compared to the untreated TiLV-infected cells (5.24 ± 0.08) (Figure 5). Furthermore, pretreatment of the TiB cells with a high PD concentration significantly reduced the TiLV concentrations at 1 dpi (4.57 ± 0.01), 3 dpi (4.73 ± 0.08) and 7 dpi (4.85 ± 0.11) compared to the positive TiLV-infected cells (4.82 ± 0.17, 5.24 ± 0.08, 5.45 ± 0.08, respectively) (Figure 5).

**Figure 6:**
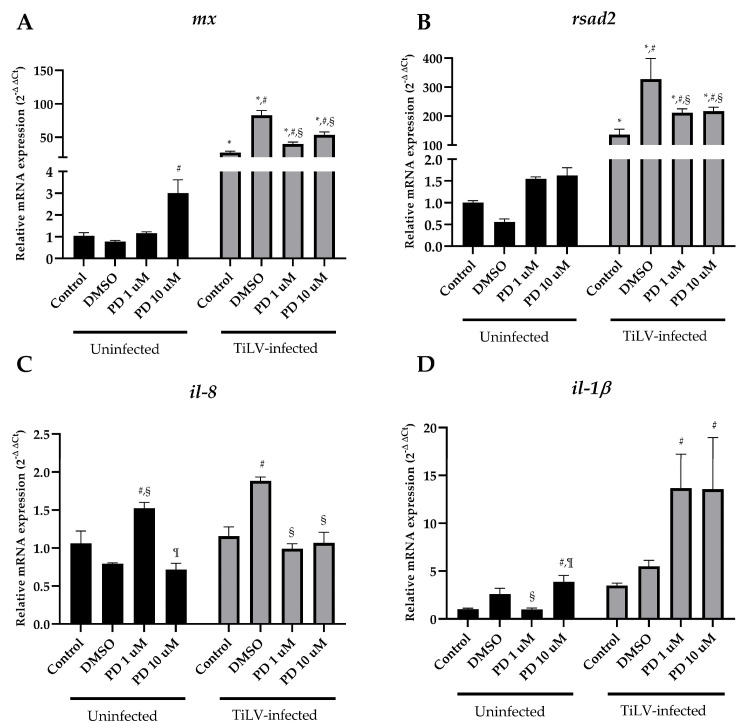
Relative expression of immune-related genes in TiB cells treated with TiLV and PD (**A**) *mx*, (**B**) *rsad2*, (**C**) *il-8* and (**D**) *il-1β* genes in uninfected and TiLV-infected TiB cells following four conditions: control cells, 1% DMSO and PD at 1 and 10 μM (n = 3 each). The data are demonstrated as means ± SEMs. * *p* < 0.05 vs. uninfected cells, # *p* < 0.05 vs. control, § *p* < 0.05 vs. DMSO, ¶ *p* < 0.05 vs. PD 1 µM.

Moreover, the expressions of the immune-related genes were investigated in the TiB cells following pretreatment with PD and TiLV. Remarkably, the TiLV infection significantly upregulated the *mx* (82.93 ± 7.08) and *rsad2* (327.57 ± 71.34) genes compared to the DMSO-treated TiLV-infected TiB cells (0.78 ± 0.05 and 0.56 ± 0.07, respectively) (Figure 6A,B). However, this upregulation was successfully suppressed by pretreatment with both low (40.06 ± 2.67 and 211.43 ± 13.78, respectively) and high (53.64 ± 4.41 and 216.97 ± 13.62, respectively) concentrations of PD. The expression of *il-8* and *il-1β* in the TiB cells also displayed distinct alternations following TiLV infection and PD treatment (Figure 6C,D). Interestingly, the expression of *il-8* in the non-infected TiB cells (1.06 ± 0.16) was elevated when pretreated with low PD (1.52 ± 0.08), while low (0.99 ± 0.07) and high (1.07 ± 0.14) concentrations of PD downregulated *il-8* in the TiLV-inoculated groups when compared with the DMSO-treated cells (1.89 ± 0.05) (Figure 6C). Moreover, the combinations of either low (13.68 ± 3.56) or high (13.56 ± 539) concentrations of PD with TiLV infection resulted in the marked upregulation of *il-1β* in the TiB cells compared to the DMSO-treated TiLV-infected cells (5.51 ± 0.61).

## 4. Discussion

The widespread prevalence of TiLV and its impact on the tilapia aquaculture industry, as well as the absence of a commercial vaccine and effective disease control measures, have highlighted the need to understand the basic mechanisms underlying the processes by which the virus causes disease in fish. One of the interesting cascades of host cell responses following the viral infection mechanism is the MAPK/ERK pathway. In this study, we investigated the involvement of MAPK/ERK associated with TiLV infection dynamics and piscine cell responses. We discovered that the MAPK/ERK pathway is necessary for viral replication and the immune response of host cells.

Our investigation revealed that TiLV elicits diverse responses in two distinct cell lines, namely, those originating from snakehead fry (E-11) and tilapia brain (TiB). The E-11 cells in our study displayed cytotoxicity and a significant increase in viral copies at 48 hpi, which is consistent with that in prior studies [5,16]. However, ERK phosphorylation in the E-11 cells did not change during TiLV infection, which suggests that the mechanism that causes cell death in E-11 cells may not utilise this pathway. Previous studies have suggested that the cytotoxic impact of TiLV infection in E-11 cells may be associated with mitochondrial dysfunction [6]. In contrast, the TiB cells in the current study did not exhibit cytotoxicity until 72 hpi, and p-ERK was suppressed at 24 hpi, which indicates a potential link between early TiLV infection and the suppression of the MAPK/ERK pathway in TiB cells, as previously discovered in transcriptomic studies [36]. The discrepancy between the MAPK/ERK responses of the two cell lines suggests that TiLV may have different tropism or entry mechanisms in different cell lines.

Previous studies have demonstrated that TiLV can enter E-11 cells as early as 1 hpi via a cholesterol-dependent pathway [37]. However, the mechanism of TiLV entry in TiB cells and other cell lines is not well understood. It has been shown that many viruses can activate the MAPK/ERK cascade during the early stages of infection to facilitate viral entry, replication and host-immune responses [27,29,30,31,32,33]. In contrast, certain viruses, such as the Ebola virus, have been shown to suppress ERK2 (p42) signals during infection, thus leading to the inhibition of cellular functions and the excessive production of proinflammatory cytokines [38]. These findings highlight the need for further research to gain a deeper understanding of the molecular interactions between TiLV and its host cells.

In our study, the use of the MEK inhibitor PD0325901 effectively suppressed TiLV replication in the TiB cells, as shown by the reduction in the viral copies at 24 hpi. This finding is in concurrence with the observation that TiLV infection impedes the phosphorylation of ERK in TiB cell lines. These results suggest that the MEK signalling pathway is crucial for TiLV replication and survival, a phenomenon observed in other viruses [27,29,30,32,33]. The requirement of the MEK1/2-ERK1/2 cascade for viral replication is well-established [28]; for example, the inhibition of MEK signalling by U0126 inhibits the spread of the borna disease virus [39,40]. Similarly, the suppression of MEK phosphorylation by PD098059 has been found to be associated with a reduction in influenza A, hepatitis C and Visna virus numbers [32,41,42]. Furthermore, MEK1/2-ERK1/2 can promote the activity of MAPK-interacting kinase 1 (MNK1) and ribosomal S6 kinase (RSK), which are associated with viral protein translation [43,44]. Collectively, the current findings demonstrate that TiLV, like other viruses, selectively targets the ERK signalling pathway to enhance its replication in TiB cells. Targeting this pathway could therefore represent a potential strategy for developing antiviral therapies against TiLV.

As parts of the antiviral responses, the *mx* and *rsad2* genes in the TiB cells in our study were significantly upregulated in response to TiLV infection at 24 hpi, as has previously been described in various organs, such as the liver, spleen, gills and intestines, following TiLV infection in tilapia [14,45,46,47] and the infection of different fish species by other viruses [48]. In addition, the suppression of MEK with PD0325901 could further attenuate *mx* and *rsad2* upregulation and TiLV concentrations. This finding indicates that TiLV targets the ERK signalling pathway to promote its replication in TiB cells. Hence, inhibiting this pathway with PD0325901 may block the ability of the virus to evade the host antiviral response, thus resulting in the suppression of viral replication. It is worth noting, however, that the suppression of antiviral gene expression could be due to a reduction in viral replication rather than directly due to MEK inhibition. Nevertheless, the same treatment did not affect the genes regulating the proinflammatory cytokines *il-8* and *il-1β*, which suggests that the MEK pathway may play a specific role in the antiviral response but not the proinflammatory process during early infection. It is possible that the activation of proinflammatory cytokine genes may occur at a later stage of TiLV infection, as has been observed in previous studies [14,47]. This highlights the multifaceted nature of host cell responses to viral infections, with different signalling pathways involved in different aspects of the cellular response. The MEK1/2-ERK1/2 pathway is known to activate the production of proinflammatory cytokines, which are crucial for coordinating immune responses against viral infections. For example, *Streptococcus agalactiae* infection in tilapia stimulates the raf-MEK1/2-ERK1/2 pathway, which leads to T-cell proliferation and the production of granzyme and interferon gamma (IFN-γ) [25,49,50]. Further research is necessary to gain a deeper understanding of how the MEK pathway regulates the antiviral response and how the virus evades this mechanism.

**Figure 7:**
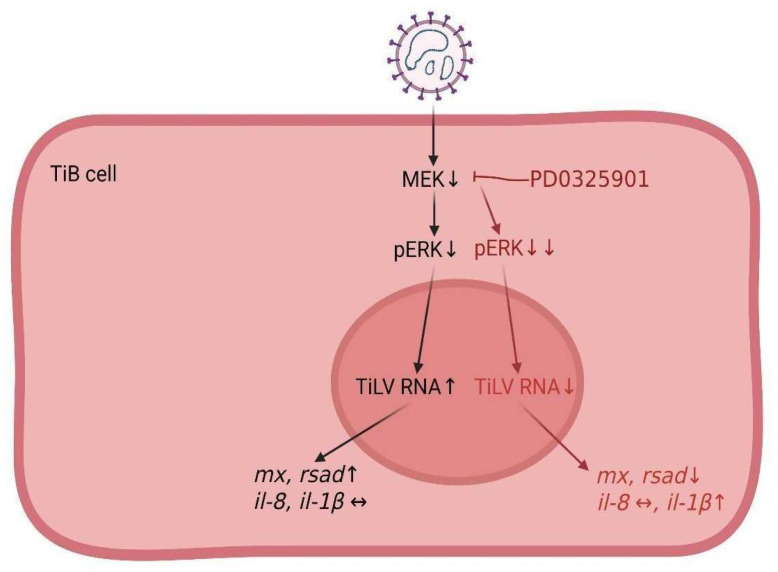
A schematic diagram illustrating the involvement of MEK/ERK signalling pathways and host-cell immune-related genes during early TiLV infection (in black). The importance of MAPK/ERK signaling in TiLV infection is elucidated by treating the cells with PD 0325901 (in red).

An overview of the current knowledge related to the stimulation of MAPK/ERK signalling in tilapia cells infected with TiLV is presented in Figure 7, in which the virus suppresses this signalling, thus leading to an increase in virus numbers. The use of the MEK inhibitor PD0325901 provided insights into the mechanism TiLV utilises during its replication. A reduction in the viral copies was consistently observed with a reduction in the antiviral genes of the host cells. These results underscore the importance of the ERK signalling pathway in the antiviral response of tilapia cells and support the potential targeting of this pathway as a therapeutic strategy.

## 5. Conclusions

Overall, the results of our study demonstrate the crucial role of MAPK/ERK signalling in the replication of TiLV and the host cell antiviral response during infection. Understanding the cellular mechanisms by which host cells respond to viral infections can facilitate the development of effective methods for preventing and controlling viral infections in animals. These findings provide important insights into the cellular and molecular mechanisms of TiLV infection, which may be valuable in the development of novel strategies to limit the spread of TiLV in the tilapia farming industry. Future studies should aim to elucidate the complex interplay between TiLV and the host cell signaling pathways as well as identify additional targets for therapeutic intervention.

## Data Availability

The data that support the findings of this study are available from the corresponding author upon reasonable request.

## Supplementary Materials

The following supporting information can be downloaded at: https://www.mdpi.com/article/10.3390/v15040900/s1, Table S1: Forward and reverse primers used in this study.
