# Supplementary material for: The Modulation of Immune Responses in Tilapinevirus tilapiae-Infected Fish Cells through MAPK/ERK Signalling
Source: Viruses. 2023 Mar 31;15(4):900. doi: 10.3390/v15040900 (PMC10144228; doi:10.3390/v15040900)
Supplement: Supplementary file 1 [file viruses-15-00900-s001.zip › viruses-2298854-supplementary.pdf]

Table S1: Forward and reverse primers used in this study

Genes	Primer Sequence (5′ – 3′)	Amplicon size (bp)	Accession number
TiLV	F: CTGAGCTAAAGAGGCAATATGGATT<br>R: CGTGCGTACTCGTTCAGTATAAGTTCT	112	KU751816
ß-actin	F: GTGGGTATGGGTCAGAAAGAC<br>R: GTCATCCCAGTTGGTCACAATA	111	XM003443127
IL-1b	F: TGGAGGAGGTGACGGATAAA<br>R: GGTGTCGCGTTTGTAGAAGA	86	XM031731335
IL-8	F: TCGCCACCTGTGAAGGCA<br>R: GCAGTGGGAGTTGGGAAGAAT	116	NM001279704
mx	F: ACCCTTGAGCTGGTGAATCA<br>R: ATCCTGAGTGAATGCGGTCA	174	XM003442686
rsad2	F: ATCAACTTCTCTGGCGGA<br>R: AGATAGACACCATATTTCTGGAAC	161	XM003453237
